# Medicaid expansion, dental visits and expenditures in veterans, older adults, and the foreign-born

**DOI:** 10.1038/s41598-025-03964-y

**Published:** 2025-06-04

**Authors:** Adejare Atanda, McKing I. Amedari, Israel T. Agaku, Lenwood W. Hayman, Mian B. Hossain

**Affiliations:** 1 School of Community Health and Policy, Morgan State University, Baltimore, MD USA; 2 RAND Corporation, Arlington, VA USA; 3 John D. Bower School of Population Health, University of Mississippi Medical Center, Jackson, MS USA; 4 Department of Oral Health Policy, Harvard School of Dental Medicine, Boston, MA USA

**Keywords:** Dental health services, Dental visits, Dental costs, Health policy, Medicaid, Affordable care act, Veterans, Older adults, Foreign-born, Epidemiology, Outcomes research, Dentistry, Health policy, Public health, Quality of life

## Abstract

We assessed the relationship between implementation of the Patient Protection and Affordable Care Act (ACA), indicated as a state expanding Medicaid, dental visits, and costs in U.S. military veterans, older adults ≥ 65 years, and foreign-born individuals. Using the 2012–2016 Medical Expenditure Panel Surveys for a secondary data analysis, logistic and two-part regressions were used to model dental visits and costs as a function of key explanatory variables. Differences-in-differences estimates compared changes in dental visits and costs in Medicaid expansion to non- expansion states. Post-Medicaid expansion and ACA implementation, the foreign-born in Medicaid expansion states had higher odds of a dental visit compared to those in non-Medicaid expansion states (OR = 1.17, 95% CI, 1.05–1.32) in adjusted analysis. While they spent $31 more than the predicted per capita expenditure of $395, this increase was not statistically significant. The changes in dental visits and expenditures for veterans and older adults ≥ 65 years were not statistically significant. These findings suggest that the ACA, through expansion of Medicaid programs has improved access for the foreign-born while controlling costs. Health care reform initiatives like Medicaid expansion can enhance access to dental care and help mitigate the economic and access to care barriers vulnerable population groups like the foreign-born face.

**Supplementary Information:**

The online version contains supplementary material available at 10.1038/s41598-025-03964-y.

## Introduction

Access to public dental-insurance coverage and adult dental benefits are key factors affecting dental care use. The cost of dental services is also a major barrier to the receipt of dental services across the life span; cost is the most common reason among working-age adults for not seeking dental care^1,2^.

Three groups represent key subpopulation segments from a policy perspective. U.S. military veterans are a special subgroup because of their varied health insurance coverage (TRICARE/VA, Medicaid, and Medicare) that makes their pattern of healthcare use different from other population groups^3,4^. Two in five veterans describe their oral health as fair or poor^5,6^. Older adults are a large and growing segment of the U.S. population, projected to surpass children in population size by 2034^7^. While accounting for only 13% of the population, older adults, 65 + years consume over 34% of national health expenditures, with 1 in 6 older adults reporting untreated caries^8^. Research into the foreign-born is important as migrants are often uninsured and experience barriers to healthcare utilization, resulting in high prevalence of periodontal disease and dental caries – 51% and 38% respectively^9–11^.

Oral health is an important part of overall health and well-being, yet the burden of oral disease is high and disparate. Almost all (92%) U.S. adults aged 20 to 64 years have experienced dental caries in their permanent teeth with unmet need (untreated caries) at 26%^5^. In its report titled *Oral Health in America* released in 2021, the U.S. Department of Health and Human Services described an epidemic of oral diseases affecting the most vulnerable, such as poor children, the elderly, veterans, and many members of racial and ethnic minority groups^12,13^. Expanding Medicaid to a broader population, including its resulting benefits in access and affordability to care and reduced financial barriers to care, leads to better health outcomes for low-income individuals^14^. Moreover, there has been increasing awareness of the need for renewed commitment and concerted policy efforts at all levels to tackle the perennial barriers to oral health access. In 2011, the Committee on Oral Health Access to Services of the Institute of Medicine and the National Research Council at the National Academies articulated the potential for the Patient Protection and Affordable Care Act of 2010 (ACA) to improve access to care for all and reduce disparities in health care and health^15^.

Although cost containment is a key ingredient of any health-reform effort, early effects seen after the ACA was implemented included both increased healthcare utilization and rising costs. This was in part due to pent-up demand; more than 20,000,000 previously uninsured Americans suddenly had healthcare either through the exchanges or Medicaid expansion^1,16^. The link between increased healthcare use and costs seen after the implementation of the ACA is poorly understood and especially unclear for dental visits and associated costs because it is understudied. While past research has examined the impact of Medicaid expansion on disparities in use of healthcare services in low-income adults, little is known about the impact among other vulnerable populations with unique dental needs and access challenges such as U.S. military veterans, older adults ≥ 65 years, and foreign-born individuals^14,17–19^. The three groups benefited from Medicaid-related expansion of adult dental benefits when states expanded Medicaid under the ACA.

Existing studies related to this topic use examine dental visits only in socioeconomic subgroups – age, education, race^17,20^. We build on this literature by examining different subpopulation groups (veterans, older adults, and foreign-born individuals), using a different survey data, and further, we extend our work to include a cost analysis using econometric methods. To inform public health policy and programs aimed at improving access to dental care among these subgroups, we explored the relationship between healthcare reform changes, dental visits, and dental costs in veterans, older adults, and the foreign-born. The healthcare reform change of interest was ACA implementation represented as Medicaid expansion. We compared changes in dental visits and costs for veterans, older adults, and the foreign-born living in states that expanded Medicaid to those living in states that did not. We hypothesized that Medicaid expansion is associated with increased dental visits and reduced costs in veterans, older adults, and the foreign-born.

Although the data used in this study are relatively old, covering 2012 to 2016, the findings are relevant to ongoing policy discussions and important for decisions that policymakers will have to make such as the prioritization of immigrants over American’s. At a time when federal funding for dental benefits under Medicaid, a program expanded under the ACA is threatened, our study strengthens the evidence base that healthcare policy changes has an impact on dental-care access and costs. Our results may be used to inform national policy to reevaluate existing public dental programs, and development of better targeted interventions for veterans, older adults, the foreign-born, and other at-risk groups.

## Results

### Descriptive results

There were 94,853 participants in the 2012–2016 MEPS and NHIS analytic sample. In total, 69% of U.S. adults were from a state where the ACA was implemented (i.e., Medicaid expansion states), while 75% and 25% respectively of those in expansion states and those in non-expansion states had a dental visit. In addition, 54% were female, 83% were aged 18–64, 43% were White and non-Hispanic, 41% had education level higher than high school, 59% were nonpoor with income > 200% of the FPL, 38% lived in the southern region of residency, 48% were married, 92% were nonveterans, 72% were U.S.-born, 93% were dentate, and 63% had no dental insurance (Table 1).

**Table 1:** Descriptive statistics, MEPS 2012–2016.

	Total	No dental visits	Dental visits
Study sample	*n* = 94,853	*n* = 62,483	*n* = 32,370
Study population	*n* = 184,655,794		
Was medicaid expansion adopted?			
Not adopted	31% (29,352)	32% (21,139)	25% (8,213)
Adopted	69% (65,501)	68% (41,344)	75% (24,157)
Sex			
Male	46% (43,953)	51% (30,379)	44% (13,574)
Female	54% (50,900)	49% (32,104)	56% (18,796)
Age			
18–64 years	83% (78,825)	83% (53,070)	78% (25,755)
≥ 65 years	17% (16,028)	17% (9,413)	22% (6,615)
Race and ethnicity			
Hispanic	29% (27,749)	20% (21,305)	10% (6,444)
White (non-Hispanic)	43% (40,560)	59% (22,162)	77% (18,398)
Black (non-Hispanic)	20% (19,194)	15% (14,389)	8% (4,805)
Asian (non-Hispanic)	8% (7,350)	6% (4,627)	5% (2,723)
Educational level			
Less than high school	23% (21,349)	19% (16,979)	9% (4,370)
High school	36% (34,523)	41% (24,669)	29% (9,854)
More than high school	41% (38,981)	40% (20,835)	62% (18,146)
Family income as % of poverty line			
Poor (< 100% )	19% (17,590)	15% (13,983)	7% (3,607)
Near poor (100–199%)	22% (21,092)	22% (16,221)	11% (4,871)
Nonpoor (> 200% )	59% (56,171)	63% (32,279)	82% (23,892)
Region of residency			
Northeast	16% (15,341)	17% (9,594)	20% (5,747)
Midwest	19% (17,732)	19% (10,411)	24% (7,321)
South	38% (36,006)	41% (26,008)	32% (9,998)
West	27% (25,774)	22% (16,470)	24% (9,304)
Marital status			
Married	48% (45,433)	48% (27,601)	59% (17,832)
Unmarried (separated, widowed, divorced)	20% (19,370)	21% (13,187)	18% (6,183)
Never married	32% (30,050)	31% (21,695)	23% (8,355)
Veteran status			
Nonveteran	92% (88,972)	93% (58,975)	92% (29,997)
Veteran	8% (5,881)	7% (3,508)	8% (2,373)
Nativity status			
U.S.-born	72% (68,520)	80% (42,997)	87% (25,523)
Foreign-born	28% (26,333)	20% (19,486)	13% (6,847)
Dentate status			
Nondentate	7% (6,458)	10% (5,380)	3% (1,078)
Dentate	93% (88,395)	90% (57,103)	97% (31,292)
Dental insurance status			
Has dental insurance	37% (34,842)	36% (18,547)	54% (16,295)
No dental insurance	63% (60,011)	64% (43,936)	46% (16,075)

### Bivariate analysis results

Chi-square tests were used to examine the relationship between each independent variable and dental visits in bivariate analysis. Results were statistically significant for all variables at *p* < .05 (Table 2).

**Table 2:** Bivariate analysis between independent variables and dental visits, MEPS 2012–2016.

	Total	No dental visits	Dental visits	Chi-square	*P*-value*
Study sample	*n* = 94,853	*n* = 62,483	*n* = 32,370		
Study population (weighted)	*n* = 184,655,794				
Was Medicaid expansion adopted?				28.9	< 0.001
Not adopted	31% (29,352)	63% (21,138)	37% (8,213)		
Adopted	69% (65,501)	56% (41,345)	44% (24,157)		
Sex				282.65	< 0.001
Male	46% (43,953)	62% (30,379)	38% (13,574)		
Female	54% (50,900)	55% (32,104)	45% (18,796)		
Age (categories based on 65)				85.04	< 0.001
Age 18–64	83% (78,825)	60% (53,070)	40% (25,755)		
≥ 65 years	17% (16,028)	52% (9,413)	48% (6,615)		
Race and ethnicity				143.85	< 0.001
Hispanic	29% (27,749)	73% (21,305)	27% (6,444)		
White (non-Hispanic)	43% (40,560)	52% (22,162)	48% (18,398)		
Black (non-Hispanic)	20% (19,194)	72% (14,389)	28% (4,805)		
Asian (non-Hispanic)	8% (7,350)	61% (4,627)	39% (2,723)		
Educational level				407.97	< 0.001
Less than high school	23% (21,349)	75% (16,979)	25% (4,370)		
High school	36% (34,523)	66% (24,669)	34% (9,854)		
More than high school	41% (38,981)	47% (20,835)	53% (18,146)		
Family income as % of poverty line				307.1	< 0.001
Poor (< 100%)	19% (17,590)	76% (13,983)	24% (3,607)		
Near poor (100–199%)	22% (21,092)	73% (16,221)	27% (4,871)		
Nonpoor (> 200%)	59% (56,171)	52% (32,279)	48% (23,892)		
Region of residency				33.2	< 0.001
Northeast	16% (15,341)	55% (9,594)	45% (5,747)		
Midwest	19% (17,732)	53% (10,411)	47% (7,321)		
South	38% (36,006)	64% (26,008)	36% (9,998)		
West	27% (25,774)	56% (16,470)	44% (9,304)		
Marital status				147.06	< 0.001
Married	48% (45,433)	53% (27,601)	47% (17,832)		
Unmarried (separated, widowed, divorced)	20% (19,370)	62% (13,187)	38% (6,183)		
Never married	32% (30,050)	65% (21,695)	35% (8,355)		
Veteran status				12.03	0.001
Nonveteran	6% (5,881)	58% (58,975)	42% (29,997)		
Veteran	94% (88,972)	55% (3,508)	45% (2,373)		
Nativity status				144.37	< 0.001
U.S.-born	72% (68,520)	56% (42,997)	44% (25,523)		
Foreign-born	28% (26,333)	68% (19,486)	32% (6,847)		
Dentate status				459.11	< 0.001
Nondentate	7% (6,458)	82% (5,380)	18% (1,078)		
Dentate	93% (88,395)	56% (57,103)	44% (31,292)		
Dental insurance status				43.21	< 0.001
Has dental insurance	37% (34,842)	48% (18,547)	52% (16,295)		
No dental insurance	63% (60,011)	66% (43,936)	34% (16,075)		

Ref stands for reference category that is being compared. * Statistically significant *P*-values for the Chi-square tests are set at *P* < .05.

### Dental visits after medicaid expansion (ACA implementation) among all U.S. adults

Main results for all time periods provide unadjusted, adjusted, and DiD results. In unadjusted regressions all variables were statistically significantly associated with dental visits in the unadjusted model (*see Model 1*, Table 3). Participants in Medicaid expansion states had 36% higher odds of having a dental visit compared to those in non-expansion states (unadjusted OR = 1.36; 95% CI: 1.21, 1.52; *p* ≤ .001). In multiple regression, all variables, except Medicaid expansion were statistically significantly associated with dental visits in the fully adjusted model (*see Model 2*, Table 3). In the DiD analysis, participants in expansion states had 9% higher odds of having a dental visit compared to participants in non-expansion states (*a*OR = 1.09; 95% CI: 1.03, 1.16; *p* = .005).

**Table 3:** Unadjusted & adjusted analysis: odds ratio & difference-in-difference estimates for dental Visits, MEPS 2012–2016.

	Unadjusted - model 1		Fully adjusted - model 2**	
Variables	OR^#^	95% CI^$^	OR^#^	95% CI^$^
DiD	-	-	1.09*	1.03, 1.16
Medicaid expansion				
Not adopted	Ref			
Adopted	1.36*	1.21, 1.52	0.98	0.87, 1.11
Sex				
Male	Ref			
Female	1.36***	1.31, 1.41	1.46*	1.40, 1.52
Age				
Age 18–64	Ref			
≥ 65 years	1.40*	1.31, 1.49	1.94*	1.80, 2.08
Race and ethnicity				
Hispanic	Ref			
White (non-Hispanic)	2.56*	2.34, 2.81	1.75*	1.60, 1.92
Black (non-Hispanic)	1.06	0.95, 1.19	0.98	0.88, 1.08
Asian (non-Hispanic)	1.78*	1.58, 2.01	1.13*	1.01, 1.27
Educational level				
Less than high school	Ref			
High school	1.56*	1.44, 1.68	1.05	0.97, 1.13
More than high school	3.43*	3.15, 3.72	1.89*	1.73, 2.06
Family income as % of poverty line				
Poor (< 100%)	Ref			
Near poor (100–199%)	1.16*	1.06, 1.27	0.96	0.88, 1.06
Nonpoor (> 200%)	2.96*	2.73, 3.20	1.64*	1.52, 1.77
Region of residency				
Northeast	Ref			
Midwest	1.12	1.00, 1.25	1.07	0.96, 1.18
South	0.68*	0.62, 0.76	0.76*	0.67, 0.85
West	0.97	0.86, 1.10	1.07	0.97, 1.18
Marital status				
Married	Ref			
Unmarried (separated, widowed, divorced)	0.70*	0.66, 0.75	0.86*	0.80, 0.91
Never married	0.60*	0.57, 0.64	0.87*	0.82, 0.93
Veteran status				
Nonveteran	Ref			
Veteran	1.16*	1.07, 1.25	1.08	0.99, 1.18
Nativity status				
U.S.-born	Ref			
Foreign-born	0.60*	0.56, 0.64	0.89*	0.83, 0.96
Dentate status				
Nondentate	Ref			
Dentate	3.51*	3.10, 3.96	3.58*	3.15, 4.06
Dental insurance status				
Has dental insurance	Ref			
No dental insurance	0.48*	0.46, 0.51	0.60*	0.56, 0.64

Ref stands for reference category that is being compared.

*Statistically significant odds ratios are set at *P* < .05. # OR: Odds ratios. $ CI: Confidence interval at the 95% level. ** Model 2 was adjusted for demographics (age, race/ethnicity, and marital status), socioeconomic level (education and poverty levels), region of residency, veteran, nativity, dentate and dental insurance status.

### Dental visits after medicaid expansion (ACA implementation) among indicated subgroups (veterans, older adults ≥ 65 years, and foreign-born)

Table 4 provides the ORs and CIs for the three subpopulations of interest: veterans, older adults age ≥ 65 years, and the foreign-born. The DiD estimator was only significant for the foreign-born. The foreign-born in Medicaid expansion states had 17% higher odds of having a dental visit compared to their counterparts in non-expansion states (*a*OR = 1.17; 95% CI: 1.05, 1.32; *p* ≤ .01).

**Table 4:** Difference-in-difference results for dental visits: stratification by veteran status, age group and foreign-born status, MEPS 2012–2016.

	Veterans**		Older adults ≥ 65 yrs.**		Foreign-born**	
Variables	OR^#^	95% CI^$^	OR^#^	95% CI^$^	OR^#^	95% CI^$^
DiD	0.97	0.76, 1.23	1.04	0.90, 1.21	1.17*	1.05, 1.32
Medicaid expansion						
Not adopted	Ref					
Adopted	1.14	0.88, 1.47	1.03	0.86, 1.24	0.89	0.71, 1.13
Sex						
Male	Ref					
Female	1.27	0.96, 1.69	1.25*	1.11, 1.41	1.47*	1.36, 1.59
Age						
Age 18–64	Ref					
≥ 65 years	2.10*	1.72, 2.56	-	-	1.66*	1.43, 1.93
Race and ethnicity						
Hispanic	Ref					
White (non-Hispanic)	1.25	0.92, 1.71	1.84*	1.48, 2.29	1.96*	1.61, 2.38
Black (non-Hispanic)	0.87	0.61, 1.25	0.74*	0.58, 0.94	1.01	0.84, 1.21
Asian (non-Hispanic)	1.28	0.76, 2.16	0.95	0.72, 1.24	1.16*	1.02, 1.32
Educational level						
Less than high school	Ref					
High school	2.96*	1.78, 4.94	1.70*	1.43, 2.02	1.24*	1.09, 1.42
More than high school	5.42*	3.31, 8.88	3.52*	2.91, 4.25	1.79*	1.58, 2.02
Family income as % of poverty line						
Poor (100% < )	Ref					
Near poor (100–199%)	0.72	0.51, 1.03	0.99	0.79, 1.26	1.14	0.97, 1.33
Nonpoor (> 200%)	1.25	0.92, 1.69	1.61*	1.30, 1.99	1.61*	1.40, 1.85
Region of residency						
Northeast	Ref					
Midwest	1.08	0.80, 1.46	0.98	0.84, 1.15	1.10	0.90, 1.35
South	0.98	0.68, 1.41	0.75*	0.63, 0.89	0.75	0.57, 0.96
West	1.27	0.92, 1.75	1.07	0.92, 1.26	1.01	0.84, 1.21
Marital status						
Married	Ref					
Unmarried (separated, widowed, divorced)	0.67*	0.54, 0.83	0.75*	0.66, 0.85	1.10	0.97, 1.26
Never married	0.61*	0.44 0.84	1.07	0.64, 1.15	0.93	0.83, 1.05
Veteran status						
Nonveteran	Ref					
Veteran	-	-	0.90	0.77, 1.06	1.52*	1.08, 2.14
Nativity status						
U.S.-born	Ref					
Foreign-born	1.12	0.76, 1.64	0.88	0.70, 1.11	-	-
Dentate status						
Nondentate	Ref					
Dentate	4.74*	3.50, 6.44	5.96*	4.93, 7.20	3.07*	2.33, 4.05
Dental insurance status						
Has dental insurance	Ref					
No dental insurance	0.71*	0.60, 0.83	0.57*	0.49, 0.66	0.49*	0.43, 0.55

Ref stands for reference category that is being compared.

*Statistically significant odds ratios are set at *P* < .05. # OR: Odds ratios. $ CI: Confidence interval at the 95% level. ** These stratified models were adjusted for demographics (age, race/ethnicity, and marital status), socioeconomic level (education and poverty levels), region of residency, veteran, nativity, dentate and dental insurance status.

### Dental expenditures after medicaid expansion: two-part regression model results

Predicted unadjusted and adjusted expenditures per capita for veterans, older adults age ≥ 65 years, and the foreign-born, by reform (Medicaid expansion) and nonreform (no Medicaid expansion) states and by reform time periods (pre-reform (2012–2013) and postreform periods (2014–2016)) are shown in Tables 5 and 6. Differences in expenditure by time period (postreform period expenditure minus pre-reform period expenditure) and DiD analysis results are also shown. None of the DiD estimates were significant in unadjusted or adjusted analysis.

**Table 5:** Unadjusted dental expenditures: difference-in-difference & two-part regression model results (USD, 95% CI), MEPS 2012–2016.

Variables	Reform states (Medicaid expansion)			Nonreform states (non-Medicaid expansion)			Difference-in-difference
	Prereform (2012–2013)	Postreform (2014–2016)	Postreform –Prereform^#^	Prereform (2012–2013)	Postreform (2014–2016)	Postreform –Prereform^#^	DiD estimate^#^ (95% CI^$^)
Veteran status							
Nonveteran	Ref						
Veteran	$37.6 (-$37.76, $112.95)	$90.69* ($40.76, $140.63)	$69.39	$91.04* ($9.23, $172.85)	$59.77* ($0.53, $119)	-$40.87	$16.99 (-$11.10, $37.09)
Age							
Age 18–64	Ref						
≥ 65 years	$78.67* ($37.14, $120.21)	$177.6* ($141.84, $213.35)	$129.30	$125.94* ($57.72, $194.16)	$112.68* ($64.89, $160.48)	-$17.33	-$0.97 (-$21.80, $20.31)
Nativity status							
U.S.-born	Ref						
Foreign-born	-$62.85* (-$117.52, -$8.18)	$-72.46* (-$107.69, -$37.23)	-$12.56	-$-41.81* (-$201.31, -$82.30	-$105 ($156.38, -$54.23)	$48.11	$19.61 (-$14.50, $44.50)

Ref stands for reference category that is being compared. * Statistically significant dental expenditures are set at *P* ≤ .05 level. $ CI: Confidence interval at the 95% level. # CPIAUCSL inflation adjusted to 2024 USD.

**Table 6:** Adjusted dental expenditures: difference-in-difference & two-part regression model results (USD, 95% CI), MEPS 2012–2016.

Variables	Reform states (Medicaid expansion)^##^			Nonreform states (nonMedicaid expansion)^##^			Difference-in-difference^##^
	Prereform (2012–2013)	Postreform (2014–2016)	Postreform–Prereform^#^	Prereform (2012–2013)	Postreform (2014–2016)	Postreform–Prereform^#^	DiD estimate^#^ (95% CI)
Veteran status							
Nonveteran	Ref						
Veteran	$19.59 (-$53.59, $92.77)	$44.45 (-$4.64, $93.55)	$32.49	$84.7* ($2.34, $167.06)	$42.88 ($37.36, $123.12)	-$54.66	$25.57 (-$4.32, $43.44)
Age							
Age 18–64	Ref						
≥ 65 years	$108.74* ($66.17, $151.30)	$198.02* ($161.13, $234.92)	$116.69	$113.01* ($48.22, $177.81)	$163.1* ($68.57, $257.62)	$65.47	$13.53 (-$11.39, $32.09)
Nativity status							
U.S.-born	Ref						
Foreign-born	-$8.14 (-$64.67, $48.39)	$3.20 (-$35.66, $42.06)	$14.82	-$58.80 (-$124.19, $6.58)	$12.21 (-$57.27, $81.69)	$92.81	$30.97 (-$4.21, $51.62)

Ref stands for reference category that is being compared. * Statistically significant dental expenditures are set at *P* < .05. $ CI: Confidence interval at the 95% level. # CPIAUCSL inflation adjusted to 2024 USD. ## These stratified models were adjusted for demographics (age, race/ethnicity, and marital status), socioeconomic level (education and poverty levels), region of residency, veteran, nativity, dentate and dental insurance status.

Total expenditures for veterans, those ≥ 65 years old, and the foreign-born were $6.52 billion, $5.20 billion, and $6.25 billion, respectively from 2012 to 2016 (Supplementary Table S4 online)^2^. Unadjusted average expenditures for veterans, those ≥ 65 years old, and the foreign-born were $3451.82, $349.71, and $408, respectively from 2012 to 2016 (Supplementary Table S4 online)^2^. Adjusted average expenditures for veterans, those ≥ 65 years old, and the foreign-born were $388.41, $350.48, and $395.36, respectively from 2012 to 2016 (Supplementary Table S4 online)^2^.

## Discussion

The overarching objective of this study was to highlight the effect of the state level implementation of the ACA among veterans, persons ≥ 65 years and the foreign born. Our analysis revealed that unlike the other target populations, the foreign-born were more likely to utilize dental services as an impact of the ACA implementation across the states consistent with Swankoski and colleagues finding that Medicaid expansion benefits vulnerable groups in a limited way (though not necessarily enough alone to ensure access to dental services)^17^. However, there was no difference in dental expenditures for any of the three sub-populations not even in the foreign-born where we have reported increased dental visits. This finding contradicts our hypothesis but is an important one because it represents the cost control piece of ACA implementation and Medicaid expansion for states that choose to do so.

Extant literature shows foreign-born U.S. residents are healthier than their U.S.-born counterparts and that healthier people tend to access or use health/medical services less^19,21–26^. Dental service utilization studies show a trend of higher rates of service utilization in the U.S.-born than the foreign-born^27^. We found the opposite with our results showing increased dental visits for the foreign-born. We think this may reflect the higher-than-average prevalence of some dental disease (periodontal disease and dental caries) reported in this group in published literature^9–11^ but our foreign-born study population was also younger (18+) versus 50 + in some of these studies. In addition, other factors such as ethnicity may be at play as acculturation effects reflecting the heterogeneity in access to care among the foreign-born^25,26,28,29^. Notably our findings demonstrate that the differences in access to care among the foreign-born may simply be attributable to living either in a Medicaid expansion state or not – If a service is available, it’s more likely to be used. This is consistent with finding that the disparities in oral health among this target population could be eliminated when insurance is accounted for and a regular source of dental care and having dental insurance are important predictors of the foreign-born’s utilization of oral health care services^9,30^.

Man Hung and colleagues had previously reported that geriatric dental expenditure witnessed a significant rise over a 20-year period^31^. However, our findings, which were over a shorter period (5 years), indicate that relative to the other target populations in our study, the total expenditure for older adults was the lowest. Our results are consistent with the findings of Charles Liu and colleagues^32^ and Kaushal and Muchomba^33^ about high expenditures among veterans (aged 65 + years) and minimal change in expenditure among foreign-born people following the implementation of the ACA. Interestingly, the high expenditures they report in veterans was driven by dental expenditures. Although there is widespread agreement that the rise of federal health policies such as the ACA substantially drives federal spending, our findings shows that there was no significant change in health expenditure with increased dental visits^34^. Kaushal and Muchomba^33^ further report that the cost implications of public health insurance for foreign-born and coverage for healthcare utilization did not impose fiscal burden on health spending. These findings provide preliminary data and guidance for formulating targeted policies for these populations and contributes to addressing controversies around this topic.

The results of this research have public health implications; firstly, the results challenge misconceptions about the fiscal burden of the impact of ACA implementation compared to states without ACA implementation. Second, states should reconsider resource allocation to public health insurance and coverage plans to include these target populations – enhanced access to preventive and timely care may increase the likelihood of improved oral health outcomes and related health outcomes. Additionally, the dissemination of these findings can foster public support for the formulation and development of more inclusive “health-in-all” policies, for example bridging the gap for underserved populations assuring equitable access to dental care.

Our findings suggest the ACA is likely to achieve a key goal of improved access to healthcare for vulnerable population groups such as the foreign-born through expansion of Medicaid programs while keeping costs under control. We have provided evidence of increased dental service utilization as a result of Medicaid expansion but the ACA’s gains in this area are likely to be reversed by efforts to completely repeal or modify its important components. Expanding Medicaid with adult dental benefits is a combination that can provide coverage for vulnerable groups who would otherwise not have access to dental care. To identify areas to strengthen adult dental benefits, policymakers, oral health advocates and supporters should consider the full continuum of coverage as this varies state to state with coverage of specific procedures, frequency of accessing service, annual service coverage monetary caps, offers of different coverage to specific groups, and the effect of multiple sources of dental coverage.

All analyses in this study reflect the common limitations of secondary use of survey data so our findings can only be generalized to noninstitutionalized U.S. civilian adults. Even though we adjusted for confounders, comparison states could differ in ways not captured by our quasi-experimental approach; therefore, the risk for residual confounding could not be completely eliminated. Crossectional survey data present challenges with respect to causal inference, and as such we do not provide conclusions regarding causal effects, only associations. Finally, despite weighting, the survey estimates may still be subject to selection bias because of differential nonresponse among different subgroups. For future inquiries into this research question, we propose the use of linked secondary data sources to follow a longitudinal design to demonstrate changes in dental service utilization and expenditures over time. We will also consider the alternative of a robust analysis of the literature to address the research question in a systematic review and meta-analysis.

## Methods

### Epidemiologic design and study population

This is a retrospective, quasi-experimental, secondary analysis of Medical Expenditure Panel Surveys (MEPS) data representative of U.S. noninstitutionalized adults aged 18 + years. Data on past-year dental visit from the full-year consolidated data files of the Household Component (HC) of MEPS were linked back to the sample adult file of the NHIS. The linkage feature of the National Health Interview Survey (NHIS) and MEPS allowed us to link 2008–2015 NHIS and 2012–2016 MEPS datasets to retrieve states Federal Information Processing Standard (FIPS) codes collected in NHIS but not MEPS. The subpopulations of interest were veterans, older adults ≥ 65 years old, and the foreign-born. Within each subpopulation, we were interested in comparing dental visits and costs between adults who lived in a state where specific ACA-related polices like Medicaid expansion were implemented versus those living in states where the policy was not implemented. Institutional Review Board (IRB) approval was requested and approved by Morgan State University. All research methods were performed in accordance with relevant guidelines/regulations as approved by Morgan State University. Informed consent was waived by the IRB committee of the Morgan State University.

### Dependent variables

We examined changes in dental visits and dental costs pre- and post-healthcare policy change. The first dependent or outcome variable was dental visits, assessed as a *binary* or dichotomous variable (i.e., yes/no). Dental visits in MEPS was a *continuous* variable (i.e., the total number of dental care visits), and included those visits to any person(s) for dental care including general dentists, dental hygienists, dental technicians, dental surgeons, orthodontists, endodontists, and periodontists in the past 12 months (Supplementary Table S1 online)^2^.

Dental expenditure was the second dependent or outcome variable analyzed. Dental expenditure was assessed as a continuous variable (i.e., total expenditure in U.S. dollars), the sum of out-of-pocket, private, Medicaid, and Medicare spending on dental services (Supplementary Table S1 online)^2^.

### Independent variable

The *key* independent or *predictor* variable was Medicaid expansion, an *indicator*, binary variable (i.e., yes/no) for ACA implementation, representing the status of state action on the Medicaid expansion decision (Supplementary Table S3 online)^2^.

### Covariates

These consisted of demographic factors such as sex, age, race/ethnicity, and marital status, and standard socioeconomic variables such as education and income/poverty levels. Education was examined as “less than high school”, “high school”, and “more than high school. Income/poverty levels is family income as % of poverty line and examined as poor (< 100%), near poor (100–199%) and nonpoor (> 200%). Also included were region of residency, veteran, nativity, dentate, and dental insurance status (Supplementary Table S3 online)^2^.

### Statistical analysis

The relationships between Medicaid expansion, the main outcome variables (dental visits and expenditures), and covariates were assessed using regression models (models specified in Supplemental Materials)^2^ estimated separately for veterans, older adults ≥ 65 years, and the foreign-born by reform time periods: pre-reform (2012–2013), reform (2014), and postreform time periods (2015–2016).

Frequencies and percentages were calculated to present a descriptive analysis. Bivariate analyses for comparison of demographic characteristics in the sample were performed using Chi-square tests for the key independent variable (Medicaid expansion), covariates, and the dependent variable (dental visits).

Unadjusted and adjusted logistic regressions (see Eq. 1 in models specified in Supplemental Materials)^2^ were performed by reform time periods; stratified by veteran status, age group, and foreign-born status, resulting in odds ratios (ORs) and 95% confidence intervals (CIs). For adjusted logistic regressions, a difference-in-differences (DiD) analysis was conducted by including a dummy variable denoting the interaction between a Medicaid expansion state during the postperiod, *post*_*i*_ *× MedExp*_*i*_, estimated separately for veterans, those ≥ 65 years old, and the foreign-born.

A two-part econometric regression model (2pm) was fit for the continuous dependent variable, dental expenditures (see Eq. 2 in models specified in Supplemental Materials)^2^. The 2pm analysis was by states (reform states vs. nonreform states), by veteran status, age group, and foreign-born status, and by reform periods as unadjusted regressions then adjusted using all covariates. Next, we used simple subtractions to calculate changes in dental care expenditures as the difference in predicted expenditures in postreform and prereform time periods for reform (Medicaid expansion) and nonreform (no Medicaid expansion) states separately using the estimated average expenditure per capita for veterans, older adults, and the foreign-born. Finally, DiD summary estimates describing the difference in predicted outcomes of the interaction terms for each observation in each of the indicated subpopulations (veterans, those ≥ 65 years, and the foreign-born) were calculated in U.S. dollars using average marginal effects. The DiD summary estimate compared those living in a reform (Medicaid expansion) state to those living in a nonreform (no Medicaid expansion) state, with the results averaged over the national sample. All survey data were weighted to adjust for the differential probabilities of selection and non-response. All reported expenditures are Consumer Price Index for All Urban Consumers: All Items in U.S. City Average (CPIAUCSL) adjusted to 2024 USD using U.S. Bureau of Labor Statistics data available by February 2025.

A detailed discussion of the statistical analytic methods used is included in Supplemental Materials^2^.

### Statement of institutional review board approval

IRB approval by Morgan State University.

## Electronic supplementary material

Below is the link to the electronic supplementary material.

Supplementary Material 1

## Data Availability

The data supporting the findings of this study are included in this published article. Raw data used for the study are publicly available from Agency for Healthcare Research and Quality (AHRQ) and National Center for Health Statistics. Clean datasets generated and/or analyzed during the current study are available from corresponding author, upon reasonable request excluding the linkage file for MEPS and NHIS data – this is solely available from AHRQ at https://meps.ahrq.gov/mepsweb/data_stats/download_data_files.jsp.
