# Supplementary material for: Medicaid expansion, dental visits and expenditures in veterans, older adults, and the foreign-born
Source: Sci Rep. 2025 Jun 4;15:19669. doi: 10.1038/s41598-025-03964-y (PMC12137644; doi:10.1038/s41598-025-03964-y)
Supplement: Supplementary file 1 — Supplementary Material 1 [file 41598_2025_3964_MOESM1_ESM.docx]

**Medicaid Expansion, Dental Visits and Expenditures in Veterans, Older Adults, and the Foreign-born**

*Adejare (Jay) Atanda DMD, DrPH, MPH^1,2^, McKing I. Amedari DMD, MPH, FMCDS^3^, Israel T. Agaku DMD, PhD, MPH^4^, Lenwood W. Hayman PhD^1^, and Mian B. Hossain MS, MHS, PhD^1^.

1School of Community Health & Policy, Morgan State University, Baltimore, MD; 2RAND Corporation, Arlington, VA; 3John D. Bower School of Population Health, University of Mississippi Medical Center, Jackson, MS; 4Department of Oral Health Policy, Harvard School of Dental Medicine, Boston, MA.

**SUPPLEMENTAL MATERIALS: Medicaid Expansion, Dental Visits and Expenditures in Veterans, Older Adults, and the Foreign-born**

**Supplementary Table S1**: *Dependent Variables*

| Measure | Description |
| --- | --- |
| *Dental visits |  |
| No | Persons without a dental visit. |
| Yes | Persons with a dental visit. |
| Dental expenditure | Total expenditure in U.S. dollars on dental services – the sum of “out-of-pocket, private, Medicaid, and Medicare” expenditures. |

*Note.* *Continuous data in MEPS, recoded into a binary or dichotomous variable for this analysis.

**Supplementary Table S2**: *Independent Variable*

| Measure | Description |
| --- | --- |
| *Medicaid expansion |  |
| No | Medicaid expansion not adopted (*untreated*). |
| Yes | Medicaid expansion adopted (*treated*). |

*Note.* *At the time of this analysis in January 2021, 38 states had adopted Medicaid expansion, and 12 states had not.(8)

**Supplementary Table S3**: *Covariates*

| Measures | Criteria |
| --- | --- |
| Sex | Male |
|  | Female |
| Age | 18–64 |
|  | >64 |
| Race and ethnicity | Hispanic |
|  | White (non-Hispanic) |
|  | Black (non-Hispanic) |
|  | Asian (non-Hispanic) |
| Educational level | Less than high school |
|  | High school/GED |
|  | More than high school |
| Poverty level | Poor |
|  | Near-poor |
|  | Nonpoor |
| Region of residency | Northeast |
|  | Midwest |
|  | South |
|  | West |
| Marital status | Married |
|  | Unmarried |
|  | Never Married |
| Veteran status | Nonveteran |
|  | Veteran |
| Nativity status | US-born |
|  | Foreign-born |
| Dentate status | Nondentate |
|  | Dentate |
| Dental insurance status | Has dental insurance |
|  | No dental insurance |

**Statistical Analysis**

The relationships between Medicaid expansion, the main outcome variables (dental visits and expenditures), and covariates were assessed using regression models as part of our retrospective, quasi-experimental, secondary data analysis.

Dental visit is modeled as follows:

*Y_ist_* = *β_0_* + *β_1_x_1_* + *β_2_x_st_* +γW+ε*_ist_*

- *Yist*: Outcome (dental visit) for individual *i* living in state *s* at time *t* (expressed as year).
- *β_0_*: State-specific fixed effects (intercept).
- *β_1_*: Regression coefficient for main predictor, Medicaid expansion status indicator for *x_1_*.
- *β_2_*: Regression coefficient for interaction term between Medicaid expansion status indicator and the postreform time period for *x_st_*. Indicators equal to 1 if the individual lived in an expansion state in reform years for the state *s* where the individual lived.
- *γ*: Vector of regression coefficients for covariates W (adjustment variables, such as sex, age, race/ethnicity, educational level, poverty status, region of residency, marital, veteran, nativity, dentate, and dental insurance status).
- ε*_ist_*: Error term
- ${Log of Odds of Dental Visits}_{it}=\beta_{0}+\beta_{1}MedExp+\beta_{2}{MedExp}_{i}\times{Post}_{i}+ \gamma W$ (1)

Dental expenditure is modeled as follows:

*Y_ist_*= *β_0_* + *β_1_x_1_* + *β_2_T_st_* + γW + *ε_ist_*

- *Yist*: Outcome for individual *i* living in state *s* at time *t* (expressed as year).
- *β0*: State-specific fixed effects (intercept).
- *β1*: Regression coefficient for the main predictor, Medicaid expansion status indicator (*x1*).
- *β2Ts*t: Regression coefficient for the interaction term between Medicaid expansion status indicator and the postreform time period (*Tst*). Indicators equal to 1 if the individual lived in an expansion state in reform years for the state *s* where the individual lived.
- *γ*: Vector of regression coefficients for covariates W (adjustment variables, such as sex, age, race/ethnicity, educational level, poverty status, region of residency, marital, veteran, nativity, dentate, and dental insurance status).
- ε*_ist_*: Error term
- ${Dental Expenditures}_{ist}=\beta_{0}+\beta_{1}MedExp+\beta_{2}{MedExp}_{i}\times{Post}_{i}+$

$\gamma W +\varepsilon_{ist}$ (2)

Both equations were estimated separately for veterans, older adults ≥ 65 years, and the foreign-born by reform time periods as described in detail under the data analysis section. Frequencies and percentages were calculated to present a descriptive analysis. Bivariate analyses for comparison of demographic characteristics in the sample were performed using Chi-square tests for the key independent variable (Medicaid expansion), covariates, and the dependent variable (dental visits). The Medicaid expansion variable and covariates were observed for statistical significance and association with dental visits (dependent variable). Cross-tabulations were used to describe the relationship between Medicaid expansion, covariates, and dental visits.

Simple logistic regressions (unadjusted) were performed because the dependent variable (primary outcome), dental visits, was dichotomous, resulting in odds ratios (ORs) and 95% confidence intervals (CIs). Multiple regression, also known as adjusted logistic regression, were performed using the same variables, resulting in ORs and 95% CIs. For both simple and multiple regressions, dummy interaction variables were included in the model as follows: an interaction of Medicaid expansion and dentate status (Medicaid expansion × dentate status), an interaction of Medicaid expansion and dental insurance status (Medicaid expansion × dental insurance), and an interaction of dentate status and dental insurance (dentate status × dental insurance).

For adjusted logistic regressions, a difference-in-differences (DiD) analysis, an analytic method to assess effect of policy changes, was conducted.(1,2,3) The DiD analysis provided summary estimates of the policy change effect across all postexpansion years. These DiD summary estimates were estimated using the same equations for the simple logistic regressions by including a dummy variable denoting the interaction between a Medicaid expansion state during the postperiod (*post_i_ × MedExp_i_*; see Equation 1). This indicator turned on starting in the year of expansion for each state. For the DiD, the treatment group was defined as the states that expanded Medicaid by January 2014, whereas the control group included the states that did not expand Medicaid by the time of this analysis in January 2021. Officially, ACA expansion was implemented by January 2014 in most of the states; however, some states that expanded before January 2014 (Michigan expanded in April 2014, New Hampshire in August 2014, Pennsylvania in January 2015, Indiana in February 2015, and Alaska in September 2015) were also classified together with the expanded states, following the procedure described by Simon et al.(4) Therefore, the DiD estimator compared the 38 Medicaid expansion states with the 12 nonexpansion states, and the researcher estimated separate equations for veterans, those ≥ 65 years old, and the foreign-born to answer the study’s research question. DiD is based on a parallel trend assumption prior to implementation of the intervention.(3) The DiD estimation is used in observational settings where exchangeability cannot be assumed between the treatment and control groups and randomization at the individual level is not possible – the use of nonequivalent control groups is what makes our study a quasi-experiment. DID relies on a less strict exchangeability assumption, i.e., in absence of treatment, the unobserved differences between treatment and control groups are the same overtime. The approach removes biases in post-intervention period comparisons between the treatment and control group that could be the result from permanent differences between those groups, as well as biases from comparisons over time in the treatment group that could be the result of trends due to other causes of the outcome.

Rather than an ordinary least square (OLS) or linear regression for the continuous, dependent variable (dental expenditures), a two-part econometric regression model (2pm) was fit. Healthcare expenditures are challenging to model because these dependent variables typically have distributions that are skewed with a large mass at zero (a “spike” of zero values) and skewness (a heavy right-hand tail), meaning most expenditures are seen in only a small proportion of the population.(5-9) These properties make OLS estimation biased and inefficient. Popular alternatives to OLS include two-part models (Manning et al., 1981; Duan et al., 1983, 1984), which model the probability of nonzero costs separately from their level conditional on nonzero costs.(10-12)

The initial 2pm analysis was done by states (reform states vs. nonreform states), by veteran status, age group, and foreign-born status, and by reform periods as simple regressions. This analysis was followed by a progressively adjusted 2pm estimated using all variables, by states (reform states vs. nonreform states), by veteran status, age group, foreign-born status, and by reform periods. Reform periods were defined as prereform (2012–2013) and postreform periods (2014–2016) in the 2pm.

The first part of the 2pm comprised of a probabilistic regression model (probit), which provided an estimate of the probability of zero versus positive expenditures. Dependent upon having a positive healthcare expenditure, a generalized linear model (GLM) with gamma distribution and a logarithmic-link function estimates the average expenditure per capita or marginal expenditure in the second part. The “margins” command was used after the 2pm to retrieve this estimate.

Using the estimated average expenditure per capita for each independent variable, the researcher used simple subtractions to calculate changes in dental care expenditures as the difference in predicted expenditures in reform and prereform time periods for reform (Medicaid expansion) and nonreform (no Medicaid expansion) states separately. Next, DiD summary estimates were calculated in U.S. dollars using average marginal effects. This DiD summary estimate was the difference in predicted outcomes of the interaction terms for each observation in each of the subpopulations (veterans, those ≥ 65 years, and the foreign-born), comparing those living in a reform (Medicaid expansion) state to those living in a nonreform (no Medicaid expansion) state, with the results averaged over the national sample. All reported expenditures are Consumer Price Index for All Urban Consumers: All Items in U.S. City Average (CPIAUCSL) adjusted to 2024 USD using U.S. Bureau of Labor Statistics data available by February 2025.

**Supplementary Table S4**: *Total and Predicted Per Capita Spending on Dental Services (USD), MEPS 2012–2016*

|  |  |  |  |
| --- | --- | --- | --- |
| Variables | Total expenditures | Unadjusted expenditures per capita | Adjusted expenditures per capita* |
| Veteran status |  |  |  |
| Non-veteran | - | - | - |
| Veteran | 6.52 B | 451.82 | 388.41 |
| Age |  |  |  |
| 18-64 years | - | - | - |
| ≥ 65 years | 5.20 B | 349.71 | 350.48 |
| Nativity status |  |  |  |
| US-born | - | - | - |
| Foreign-born | 6.25 B | 408 | 395.36 |

* Adjusted for demographics (age, race/ethnicity, and marital status), socioeconomic level (education and poverty levels), region of residency, veteran, nativity, dentate and dental insurance status. All expenditures are CPIAUCSL inflation adjusted to 2024 USD.

**References**

1. Guth M, Garfield R, Rudowitz R. The Effects of Medicaid Expansion under the ACA: Studies from January 2014 to January 2020. Kaiser Family Foundation, March. 2020;17.

2. Kino S, Kawachi I. The impact of ACA Medicaid expansion on socioeconomic inequality in health care services utilization. PloS one. 2019;13(12):e0209935.

3. Miller S, Johnson N, Wherry LR. Medicaid and mortality: new evidence from linked survey and administrative data: National Bureau of Economic Research2019.

4. Simon K, Soni A, Cawley J. The impact of health insurance on preventive care and health behaviors: evidence from the first two years of the ACA Medicaid expansions. Journal of Policy Analysis and Management. 2017;36(2):390-417.

5. Sugawara S, Wu T, Yamanishi K. A basket two-part model to analyze medical expenditure on interdependent multiple sectors. Statistical Methods in Medical Research. 2018;27(5):1585-600.

6. Buntin MB, Zaslavsky AM. Too much ado about two-part models and transformation?: Comparing methods of modeling Medicare expenditures. Journal of Health Economics. 2004 2004/05/01/;23(3):525-42.

7. Deb P, Norton EC. Modeling Health Care Expenditures and Use. Annual Review of Public Health. 2018;39(1):489-505.

8. Okunrintemi V, Valero-Elizondo J, Michos ED, Salami JA, Ogunmoroti O, Osondu C, et al. Association of Depression Risk with Patient Experience, Healthcare Expenditure, and Health Resource Utilization Among Adults with Atherosclerotic Cardiovascular Disease. Journal of General Internal Medicine. 2019 2019/11/01;34(11):2427-34.

9. Nasir K, Okunrintemi V. Association of patient-reported experiences with health resource utilization and cost among US adult population, medical expenditure panel survey (MEPS), 2010–13. International Journal for Quality in Health Care. 2018;31(7):547-55.

10. Duan, N., Manning, W. G., Morris, C. N., & Newhouse, J. P. (1983). A comparison of alternative models for the demand for medical care. *Journal of Business & Economic Statistics*, *1*(2), 115–126. <https://doi.org/10.2307/1391852>

11. Duan, N., Manning, W. G., Morris, C. N., & Newhouse, J. P. (1984). Choosing between the sample-selection model and the multi-part model. *Journal of Business & Economic Statistics*, *2*(3), 283–289.

12. Manning, W. G., Morris, C. N., Newhouse, J. P., Orr, L. L., Duan, N., Keeler, E. B., Leibowitz, A., Marquis, K. H., Marquis, M. S. & Phelps, C. E (1981). A two-part model of the demand for medical care: Preliminary results from the health insurance study. *Health, Economics, and Health Economics*, *137*, 103–123.
